# Is Electronic Health Literacy Associated with Learning Outcomes among Medical Students in the First Clinical Year?: A Cross-Sectional Study

**DOI:** 10.3390/ejihpe11030068

**Published:** 2021-08-19

**Authors:** Krittai Tanasombatkul, Kanokporn Pinyopornpanish, Chaisiri Angkurawaranon, Nida Buawangpong, Auswin Rojanasumapong, Wichuda Jiraporncharoen

**Affiliations:** Department of Family Medicine, Faculty of Medicine, Chiang Mai University, Chiang Mai 50200, Thailand; krittai.t@cmu.ac.th (K.T.); chaisiri.a@cmu.ac.th (C.A.); nida.buawangpong@cmu.ac.th (N.B.); auswin.r@cmu.ac.th (A.R.); wichuda.j@cmu.ac.th (W.J.)

**Keywords:** electronic health literacy, internet use, medical students

## Abstract

Medical students tend to use the internet as a primary resource when seeking health information. This study aims to assess the patterns of internet use, eHL level, and learning outcomes with eHL among medical students at Chiang Mai University. A cross-sectional study was conducted among 88 medical students in the first clinical year. The eHL level was determined using the Thai version of the electronic Health Literacy Scale or eHEALS. The patient case report scores were obtained representing the learning outcome. Linear regression was used to identify factors influencing their eHL level and case report scores. Students recognized the importance and usefulness of the internet. The mean eHEALS score was 33.45. There was a lower degree of agreement on questions regarding internet usage, having skills to evaluate the resources, and confidence in using health information to make health decisions. The eHEALS score had no statistically significant association with most variables and case report scores, but with the longer time of internet use (*p*-value = 0.014). Although medical students perceived that they have high eHL levels, they report lower confidence in using the information. Including critical thinking skills for electronic health information in the medical curriculum could be useful.

## 1. Introduction

In the 21st century, while chronic diseases have been escalating worldwide, the current style of training and skill set of health providers might not be adequate to manage patients with these conditions [1]. Therefore, the World Health Organization (WHO) has suggested five core competencies that the health care workforce needs for caring for patients with chronic conditions. These competencies consist of (1) patient-centered care, (2) partnering, (3) quality improvement, (4) information and communication technology, and (5) public health perspective [2]. In this digital era, as the internet becomes the major source of information, the fourth competency is important. Transformation of medical education and training to prepare the future health profession for electronic health literacy is necessary to develop competency in information and communication technology. Besides basic knowledge of the internet, healthcare professionals should be knowledgeable of how to evaluate sources of information as credible sources of information [3]. A previous study has suggested that eHealth education should start from the basic university level [4].

At present, undergraduate healthcare students usually use the internet as a primary resource when seeking health information [5]. Via this online platform, they conveniently obtain and learn many kinds of information involving various aspects of human life both for their everyday life and academic purposes. The internet encourages learning activities at any time and place in just one click [6]. However, while it provides access to a wide range of learning resources and delivered information, medical students might be overwhelmed by this large amount of information [7]. Therefore, the ability to seek, find, understand, and appraise health information from electronic resources is necessary to apply such knowledge to addressing or solving health problems and is known as electronic health literacy (eHealth literacy or eHL) [8].

eHL encompasses six core literacy skills which consist of traditional literacy, health literacy, information literacy, scientific literacy, media literacy, and computer literacy [8]. It is not static, but rather process-oriented, and evolves with the introduction of new technologies and changes in personal, social, and environmental contexts. Therefore, eHL is under influence of a variety of factors including level of education, availability and accessibility of internet, and income [9]. Having a high level of eHL is important for medical students because, during this period of life, they are developing lifelong learning skills and gathering experience in working with technology [6]. Medical students not only use the health information for taking care of their health but also learn to use this information to create care plans for their patients. Thus, a good understanding of perceptions about internet use and personal factors that influence eHL of medical students is important for medical educators to help improve their skills to use for themselves and patient care.

Several studies found that university students self-reported a lack of ability to conduct successful health-related internet searches [10,11]. In addition, studies showed that undergraduate healthcare students had low eHL skills [12,13]. Previous studies from a systematic review suggest that even if university students report that they feel comfortable with internet use, their eHL skills may still be insufficient. Poor usability of eHealth services can pose difficulties to online health information access and usage [14]. This result suggests that there is room for better understanding and improvement in education about eHL skills for university students, especially in the healthcare profession [15]. A study in South Korea was undertaken to measure nursing students’ ehealth literacy. The majority of participants claimed the Internet aided them in making health-related decisions, but only a small number of them could distinguish between high-quality and low-quality sources [16]. Similar to the findings of another study, nursing students had difficulties analyzing these sources and distinguishing between high-quality and low-quality sources despite being aware of Internet resources and browsing the Internet [17]. According to the findings of another study, pharmacy students have poor self-reported eHealth literacy and lack confidence in using technology [18]. Although many studies have focused on eHealth literacy of undergraduate healthcare students, little is known about eHealth literacy among undergraduate medical student who will be future medical professionals that interact with patients on a regular basis. A previous study among Saudi medical students shows that the students’ overall understanding of e-health was inadequate [19]. The direct study of eHL levels in Asian medical students was conducted in Vietnam medical university, which shown insufficient eHL.

Findings from a recent review show that most studies on eHL are consumer-oriented [20]. They aim to measure eHL levels by mostly using the most popular measurement called the eHealth literacy scale or eHEALS while context-oriented evidence was much less. The illustration of context-oriented focus research include effects on health behavior, ability to evaluate eHealth information, or giving suggestion for others. However, there is no study on learning outcomes.

The evidence of eHL among medical students in Asia and Thailand is still limited [16,21,22], while the use of the internet and online learning has accelerated with the COVID pandemic, the present study aimed to assess the characteristics of internet use as a source for creating patient care plans and to measure eHL levels and examine factors associated with eHL among medical students at Chiang Mai University in their first clinical year, including learning outcomes. We hypothesized that medical students’ eHL would have a substantial influence on their patient case report scores. Regarding the five core competencies by WHO on preparing a health care workforce for the 21st century, findings from this study could provide basic information on eHealth literacy of future healthcare and help guide the way to improve medical education relating to the fourth competency about using computer and technologies.

## 2. Materials and Methods

### 2.1. Study Design

A cross-sectional study was conducted involving medical students, who were in their first clinical year, rotating in the Department of Family Medicine, Chiang Mai University.

### 2.2. Participants and Setting

All fourth-year medical students rotating in the Department of Family Medicine between October 2019 and March 2020 were included. The Faculty of Medicine, Chiang Mai University is the largest medical school in the Northern part of Thailand. The Doctor of Medicine degree will be awarded after the students complete the six-year medical program. The first three years are pre-clinical years that emphasize basic knowledge regarding basic medical sciences. The students will spend more time in clinical settings during the following three clinical years (4th–6th year). During the first clinical year (4th-year medical students), the Family Medicine rotation is one of the requirements for every student. Medical students are assigned to examine patients under supervision at the Family Medicine Outpatient Clinic, which is a primary care setting in the hospital and most patients have an uncomplicated or common disease. They practice implementing their clinical knowledge in creating patient care plans by emphasizing a patient case report.

### 2.3. Procedures

The data were collected at the end of the Family Medicine rotation. After giving informed consent, each participant completed a self-administered questionnaire, which consisted of three main sections: (1) demographic data, (2) internet use information, and (3) eHL assessment. At the end of the semester, case report scores, which were representative of the learning outcomes of each participant were retrieved by the researcher.

### 2.4. Materials and Tools

Demographic data included age, gender, hometown, income per month, parent education, and parent occupation. Internet use information consisted of the usage diary, timing, and frequent use of electronic sources. Internet diary usage is the total amount of time spent on the internet, whether it is for personal or academic purposes. Students provided names of websites that they used for their health care and complete the case report. The numbers of sites were counted from the name list. 

The eight-item eHealth Literacy Scale (eHEALS) was represented as a tool to assess eHL. This is a globally validated self-reported tool to assess perceived knowledge (Question 1–5), skills (Question 6–7), and confidence (Question 8) in finding health information from electronic resources and applying it to the management of health problems. Each item is rated on a Likert scale of 1 to 5 (1 strongly disagree and 5 strongly agree). In addition to the eight items, it has two questions (A and B) apart from the eight items which rate the usefulness and importance of electronic health resources [23].

The patient case report scores in family medicine rotation represented the learning outcome. Writing a case report provides an opportunity for medical students to critically search for evidence-based practice and integrate the knowledge into patient care. Case report scores were evaluated by random instructors of the Family Medicine Department using the rubric scoring guide. The clinical skills that were evaluated from the case report included the case approach, differential diagnosis, and specific treatment plan for the patient. After examining the patient at the outpatient clinic, each student had seven days to finish a case report. The maximum score was 100 points. 

### 2.5. Statistical Analysis

All statistical analyses were performed using Stata 16 (Stata Corp, College Station, TX, USA). We described categorical variables with frequency and percentage. For continuous variables, we tested the normality of each variable by using the Shapiro–Wilk test. We present mean and standard deviation for normally distributed variables and median and IQR for non-normally distributed variables. As eHEALS has never been validated in the Thai population, we conduct factor analysis to evaluate whether the factor loading of each item was consistent with those in the derivation study [23]. The number of factors was determined using a simple structural methodological solution based on eigenvalues and the scree plot, which is generally defined when eigenvalues surpass 1.0 same as the derivation study. In order to be considered acceptably relevant, each item must have a factor loading of at least 0.4 [24,25]. Internal consistency reliability of eHEALS was presented using Cronbach’s alpha [26]. Univariable and Multivariable linear regression analysis was used to analyze the associated factors with eHEALS and the association between the eHEALS and case report scores. A *p*-value of less than 0.05 was considered statistically significant. 

## 3. Results

A total of 88 medical students were enrolled in the study. Around 56% of the students were male with a median age of 22 years (IQR = 21.22), 22% had at least one underlying disease. A large number of the students’ parents had graduated above bachelor’s degrees (76%), but 29.5% worked as healthcare providers. The mean case report score was 76.52 (SD = 11.57). All participants had a smartphone. Demographic data are described in Table 1.

Table 2 showed the median time of five hours using the internet (IQR = 3, 8). The median number of electronic sources that participants used as main sources of health information was five sources (IQR = 3, 7). Most students used UptoDate.com (accessed on 16 August 2021) and Pubmed.gov (accessed on 16 August 2021) as primary resources for writing their research. In terms of personal health care, most students use UptoDate.com and local Thai websites as primary sources.

Table 3 shown factor analysis and internal consistency. Factor analysis was conducted with a single-factor solution as expected (eigenvalue = 3.45, 43% of the variance explained). The factor loadings for the eight items ranged from 0.53 to 0.87. The factor loadings of all eHEALS items in this study were more than 0.4 and were consistent with those in the derivation study. Reliability statistic was performed on eight items, shown a fitting scale with Cronbach’s Alpha 0.83.

Table 4 showed the mean total score of eHEALS (Question 1–8) was 33.45 (SD = 3.28) out of the possible 40 with a score range from 26 to 40. Almost all the participants (95.45%) perceived that the internet was useful or very useful for making health decisions. Similarly, 97.73% felt that it was important to be able to access health-related resources. Although the majority of the participants either strongly agreed or agreed that they know about accessing health information on the internet (Question 2–3), there was a lesser degree of agreement on knowing how to use information, have skills to evaluate the resources, and confidence in using health information to make health decision (Question 5–6 and Question 8).

In univariable analysis, there was no significant association between variables and eHEALS scores, except for the long time of internet use (*p*-value = 0.013). This association was also observed in the multivariable analysis as reported in Table 5 (coefficient = 0.23; 95% CI 0.04, 0.42; *p*-value = 0.014). There was no association between the eHEALS score, and the case report score in Table 6 (coefficient = −0.53; 95% CI −1.36, 0.31; *p*-value = 0.215).

## 4. Discussion

The major findings of this study were that the medical students in their first clinical year reported the use of the internet as one of the information resources for their studies, recognized its importance and usefulness, and UpToDate was the most popular internet resource for academic report writing. Students reported overall high eHEALS scores but perceived less knowledge on how to use information, fewer skills to evaluate the resources, and less confidence in using health information to make health decisions. Longer hours spent on the internet were associated with a higher eHEALS score, while the eHEALS score showed no association with case report scores.

Students in our study reported that they found internet use to be important and useful, which is related to the previous finding from the systematic review [15]. Thus, unsurprisingly, internet use as a source for creating patient care plans was reported by students. To search for information for writing their patient case report, the medical students used electronic sources more than physical textbooks, which is congruent with a previous study [6]. One reason why students may choose electronic resources over paper textbooks is the availability and accessibility of the resources [6,27]. The characteristic of internet use was that UpToDate.com was the most commonly used online resource. UpToDate.com is a subscription-based resource that has a navigation system that allows quickly jumping to and through topics to a specific answer to a clinical question [28]. Medical students prefer summarized information sources such as UpToDate.com over primary literature searches, which make it easier to integrate the knowledge into real-life patient care [29,30]. Also, we found that Google was the most frequently used as a starting source for locating health information. Similarly, a previous study reported medical students selected Google or UpToDate to locate a piece of information needed instead of conducting a complete literature review [31]. 

eHL levels were overall high. The average eHEALS score of the medical students in this study was 33.45. This score is relatively higher than the non-communicable disease patient at the Family Medicine Outpatient Clinic of our institute, with a mean score of 28.6 points [32]. Also, previous studies from other countries reported lower scores. For example, the mean eHEALS score of Vietnam medical students was 27.03 points [22], the mean eHEALS score of Nepal interns who represented immediate past medical students was 28.44 [33], and the mean eHEALS score of students of an Iranian medical and health science university was 28.20 points [13]. There are some possible explanations for the higher eHL of medical students. As eHL is literacy focusing on health-related information, medical students with more exposure to medical and health information in the curriculum of the faculty faired better compared to other non-medical curricula. This is supported by previous studies that undergraduate students in the medical sciences had higher eHEALS scores than those in nonmedical departments [21]. Moreover, a self-directed learning process could encourage them to use technology and the internet to obtain health information. However, as eHEALS is a self-report questionnaire, students might overestimate their performance [34]. In college students, a gap between the perceived and actual eHL has been reported [15,35]. This evidence could be supported by the findings in our and the previous studies that participants rated themselves with a lower score on the questions about confidence and skills to use eHealth information [12,16]. 

No statistically significant association between variables and eHEALS score was observed in our study, except for the long time of overall internet use per day. Medical students in our study spent approximately five hours a day on the internet (overall use, not limited to the health information seeking), which is relatively higher than a previous study showing only two hours [16]. The more time they spent would allow them to look for more resources and might increase their chance to apply more evidence to practice [35]. However, the association between eHL and the case report score, which might represent the integration of knowledge obtained from eHealth resources to practice, was not observed in our study. It could have resulted from the high eHL reported by all participants. All results implied that medical students might face a large volume and variety of eHealth information. Information overload may cause errors in clinical care [7]. 

Our results could highlight the necessity of assisting medical students in enhancing their confidence in seeking and using electronic health information. Intervention to enhance their ability and skills to use electronic health information could be useful. For example, the two-week e-learning activities, which consist of topics about the reliability of the information on the internet, scientific research methods, and cautions regarding health information posted on social networking websites, can improve the eHEALS scores [36]. The E-learning or online learning platform, especially during the pandemic of COVID-19 where students cannot physically attend classes, can enhance the teaching and learning experience as it is convenient, and it allows medical students and medical instructors greater flexibility in both their location and times. Medical students can benefit from e-learning to help them adjust to a web-based medical world that is more familiar with digital health services [37]. In addition, medical educators must offer active guidance for a better approach to seek online information. For example, a training workshop about evidence-based searching techniques can raise awareness of best evidence and clinician’s justifiable self-confidence and improve the searching skills for suitable online evidence [38]. 

There were some limitations in this study. Firstly, as this study was conducted in a single medical center, the results may not apply to university students in different settings. Secondly, according to the nature of a cross-sectional study, it could not determine the temporal relationship. However, reverse causation is unlikely and cross-sectional study is a commonly used method in the literature. The study attempted to assess the association between eHL and learning outcomes with scarce evidence. We believed that a case report should be a good proxy for integrating medical knowledge into practice. The writing pattern and evaluation form were provided to reduce the difference in writing patterns and styles. However, the writing skills of the student might influence the score, even though all have been trained to write a case report before their first clinical year. Assessment of learning outcomes with different methods might provide different results. Lastly, due to the COVID-19 pandemic situation, the study schedules and rotations have been changed and interrupted. Further recruitment was stopped earlier than the expected plan; thus, leading to a smaller sample size. Further study with a larger sample size would be recommended to provide more information.

## 5. Conclusions

In the 21st century where electronics and the internet are commonly used in medical school, improving medical students’ competency in information and communication technology is necessary. The level of eHealth literacy among Thai medical students was still unknown. Our study had found that eHealth literacy levels of medical students in Thailand were relatively high, but some domains of electronic health literacy can be improved, particularly in enhancing their skills and confidence to use eHealth information. Introducing concepts of eHealth literacy early in medical school may be necessary along with training workshops to enhance evidence-based searching and appraising techniques could be useful. Even though our study found no association between eHL and learning outcome, we have added to a growing literature about using eHL skills in the medical education field. Further studies on interventions that will help develop eHealth literacy in developing countries are still needed as this should likely lead to better learning and integration of this 21st-century skill in their practice.

## Figures and Tables

**Table 1:** Characteristics of Participants.

Characteristic		Number (%)
Gender	Male	49 (55.68)
	Female	39 (44.32)
Age	Median (IQR)	22 (21, 22)
Hometown	Urban	47 (53.41)
	Rural	41 (46.59)
Income (THB)	Median (IQR)	10,000 (8000, 12,000)
Scholarship	Yes	9 (10.23)
	No	79 (89.77)
Highest parent education	Under bachelor’s degree	21 (23.86)
	Above bachelor’s degree	67 (76.41)
Parent job is Healthcare provider	Yes	26 (29.55)
	No	62 (70.45)
Underlying disease	Yes	19 (21.59)
	No	69 (78.41)
Case report scores	Mean (SD)	76.52 (11.57)

Abbreviations: SD, Standard deviation; IQR, Interquartile range; THB, Thai Bath.

**Table 2:** Internet Use Information of Participants.

Internet Use of Participants		Number (%)
Internet use hour/day	Median (IQR)	5 (3.8)
	≥5 h/day	51 (57.9)
	<5 h/day	37 (42.1)
Number of electronic sources	Median (IQR)	5 (3, 7)
Number of physical texts	Median (IQR)	3 (2, 4)
Popular electronic source for academic purpose	UptoDate	79 (25.15)
	Pubmed	71 (22.61)
	Medscape	52 (16.56)
	Clinical Keys	31 (9.82)
	WebMD	31 (9.82)
	Local Thai website	25 (7.96)
	Wikipedia	16 (5.09)
	Google scholar	9 (9.86)

Abbreviations: IQR, Interquartile range.

**Table 3:** Factor analysis and reliability scale of eHEALS.

Item	Factor Loading	
	Derivation Study	This Study
Q1.I know what health resources are available on the internet.	0.77	0.53
Q2.I know where to find helpful health resources on the internet.	0.79	0.78
Q3.I know how to find helpful health resources on the internet.	0.77	0.87
Q4.I know how to use the internet to answer my questions about health.	0.84	0.74
Q5.I know how to use the health information I find on the internet to help me.	0.81	0.73
Q6.I have the skills I need to evaluate the health resources I find on the internet.	0.72	0.66
Q7.I can tell high-quality health resources from low-quality health resources on the Internet.	0.65	0.56
Q8.I feel confident in using information from the Internet to make health decisions.	0.60	0.64
Variance accounted percent	56%	43%
Cronbach’s Alpha	0.88	0.83

**Table 4:** Average eHEALS Scores of Participants.

eHEALS Questions	Median (IQR)	Mean (SD)	No. of Participant Rating to the Score * (Row %)		
			3	4	5
A. How useful do you feel the internet is in helping you make decisions about your health?	5 (4.5)	4.55 (0.58)	4.55	35.23	60.23
B. How important is it for you to be able to access health resources on the internet?	5 (4.5)	4.67 (0.51)	2.27	28.41	69.32
Q1. I know what health resources are available on the internet.	4 (4.5)	4.21 (0.59)	9.09	60.23	30.68
Q2. I know where to find helpful health resources on the internet.	4 (4.5)	4.39 (0.59)	5.68	48.86	45.45
Q3. I know how to find helpful health resources on the internet.	4 (4.5)	4.32 (0.56)	4.55	57.95	37.50
Q4. I know how to use the internet to answer my questions about health.	4 (4.5)	4.20 (0.61)	10.23	59.09	30.68
Q5. I know how to use the health information I find on the internet to help me.	4 (4.4)	4.09 (0.61)	14.77	61.36	23.86
Q6. I have the skills I need to evaluate the health resources I find on the internet.	4 (4.4)	3.97 (0.64)	21.59	59.09	19.32
Q7. I can tell high-quality health resources from low-quality health resources on the Internet.	4 (4.5)	4.25 (0.64)	11.36	52.27	36.36
Q8. I feel confident in using information from the Internet to make health decisions.	4 (4.4)	3.98 (0.59)	18.18	64.77	17.05

Abbreviations: Q, Question number; SD, Standard deviation; IQR, Interquartile range. * No participant rated on 1–2 score.

**Table 5:** Factors Associated with eHEALS Score.

	Univariable Linear Regression Analysis			Multivariable Linear Regression Analysis		
eHEAL Score	Coefficient	95% CI	*p*-Value	Coefficient	95% CI	*p*-Value
Female	1.04	−0.34, 2.43	0.139	0.98	−0.55, 2.52	0.205
Age	−0.27	−1.25, 0.70	0.577	−0.17	1.24, 0.88	0.742
Hometown	−0.57	−1.99, 0.85	0.426	−0.37	−1.98, 1.23	0.641
Income	−0.01	−0.01, 0.15	0.875	0.02	−0.16, 0.21	0.798
Scholarship	0.24	−2.07, 2.54	0.839	0.15	−2.29, 2.61	0.899
Highest parent education	−0.72	−2.35, 0.91	0.387	−0.61	−2.35, 1.12	0.487
Parent job is Healthcare provider	0.50	−1.03, 2.03	0.517	0.49	−1.18, 2.17	0.559
Underlying disease	−0.11	−1.81, 1.59	0.898	−0.45	−2.26, 1.35	0.616
Hour of internet use/day	0.22	0.05, 0.39	0.013	0.23	0.04, 0.42	0.014
Number of devices	0.17	−0.78, 1.12	0.728	−0.05	−1.05, 0.94	0.911

Abbreviations: CI, Confidence Interval.

**Table 6:** Factor Associated with Case Report Scores.

	Univariable Linear Regression Analysis			Multivariable Linear Regression Analysis *		
Case Report Scores	Coefficient	95% CI	*p*-Value	Coefficient	95% CI	*p*-Value
Female	1.58	−3.37, 6.53	0.527	2.06	−3.13, 7.25	0.432
Age	0.77	−2.67, 4.22	0.655	0.25	−3.44, 3.93	0.895
Hour of internet use/day	−0.56	−1.18, 0.49	0.071	−0.45	−1.14, 0.23	0.192
eHEALS scores	−0.59	−1.33, 0.15	0.117	−0.53	−1.36, 0.31	0.215

Abbreviations: CI, Confidence Interval. * Adjusted by groups of students.

## Data Availability

The datasets used and/or analyzed during the current study are available from the corresponding author on reasonable request.
